# Mechanism of *YAP1* in the senescence and degeneration of endplate chondrocytes induced by intermittent cyclic mechanical tension

**DOI:** 10.1186/s13018-023-03704-w

**Published:** 2023-03-22

**Authors:** Lei Kong, Yong-Sheng Xie, Xu-Dong Ma, Yan Huang, Xi-Fu Shang

**Affiliations:** 1 Division of Life Science and Medicine, Department of Orthopedic, The First Affiliated Hospital of USTC, University of Science and Technology of China, HeFei, 230001 Anhui China; 2 Division of Life Science and Medicine, The First Affiliated Hospital of USTC, University of Science and Technology of China, HeFei, 230001 Anhui China; 3 BengBu Medical College, Bengbu, 233030 Anhui China

**Keywords:** Endplate cartilage, Degenerative, Senescence, *YAP1*, ICMT

## Abstract

**Background:**

This study aimed to investigate the potential mechanism of *YAP1* in the senescence and degeneration of endplate chondrocytes induced by intermittent cyclic mechanical tension (ICMT).

**Methods:**

According to the Pfirrmann grade evaluation classification, 30 human endplate cartilage tissues were divided into the lumbar vertebra fracture (LVF) group and lumbar disc herniation (LDH) group. Then, quantitative reverse transcription polymerase chain reaction, western blot, flow cytometry, hematoxylin–eosin staining, and senescence-associated β-galactosidase staining were performed. The difference in extracellular matrix expression between LVF and LDH endplate cartilage was detected. Second, the effect of ICMT on endplate chondrocytes degeneration was observed. Finally, the key regulatory role of *YAP1* in ICMT-induced endplate cartilage degeneration was further verified.

**Results:**

In degraded human endplate cartilage and tension-induced degraded endplate chondrocytes, the expression of *YAP1*, *COL-2A*, and *Sox9* was decreased. Conversely, the expression of *p53* and *p21* was increased. By regulating *YAP1 *in vivo and in vitro, we can achieve alleviation of ICMT-induced senescence of endplate chondrocytes and effective treatment of disc degeneration.

**Conclusions:**

ICMT could induce senescence and degeneration of endplate chondrocytes, and ICMT-induced senescence and degeneration of endplate chondrocytes could be alleviated by regulating *YAP1* expression.

## Background

Intervertebral disc degeneration (IDD) is one of the main causes of low back pain in middle-aged and older adult patients. IDD involves decreased intervertebral disc nucleus pulposus (NP) water content, intervertebral space loss, NP herniation, and secondary spinal stenosis. The decrease of endplate chondrotrophic transport is an important pathogenic factor of IDD [1, 2]. The main route of nutritional supply to the intervertebral disc is the endplate cartilage, which is the hydrated biological tissue located above and below the intervertebral disc [3, 4]. Additionally, the intervertebral disc relies on the vertebral cartilage endplate (CEP) as its main route of nutritional supply [3, 4]; therefore, blocking the vertebral CEP can induce intervertebral disc degeneration and apoptosis [5]. Furthermore, other studies using degenerative intervertebral disc specimens have shown that as individuals get older and metabolism slows down, the rates of senescence and apoptosis of intervertebral disc cells are as high as 53–73% [6]. Increased inflammation or degeneration of the endplate cartilage leads to decreased expression of type II collagen (*COL-2A*) in the intervertebral disc, which is highly correlated with IDD [7, 8]. Additionally, reports have shown that endplate cartilage degeneration is a potential cause of IDD [9–11]. Maintaining the normal function of endplate cartilage plays a decisive role in preventing IDD and senescence [12].

In the complex biomechanical environment inside and outside the body, biomechanics affect the molecules, cells, tissues, and organs of biological organisms and even the entire organic individual. Tension plays a key role in this process; particularly, endplate chondrocytes are embedded in the cartilage lacuna through the adhesion structure, generating tension in the adhesion process [13, 14]. Endplate chondrocytes sense the stretch stimulus generated by the adhered cartilage tissue and respond to external mechanical signals by regulating the extracellular matrix synthesis accordingly [15]. The Hippo-Yes-associated protein (*YAP)* pathway is composed of a group of conserved kinases, which are involved in biological processes such as cell proliferation, differentiation, and senescence. This pathway is regulated by upstream membrane protein receptors which receive extracellular growth inhibition signals. Subsequently, these receptors activate the downstream phosphorylation of a series of kinase cascades; finally, the downstream effectors phosphorylate *YAP* and *TAZ* to exert biological effects [16, 17]. *YAP* is an important transcription factor in the Hippo pathway; particularly, *YAP1* has been shown to be involved in the regulation of various cellular processes including stem cell proliferation and differentiation [18]. *YAP* is a transcription factor that regulates cell proliferation and vitality; additionally, *YAP* is closely related to senescence [19–21]. Regardless of changes in the expression of upstream elements, *YAP* requires phosphorylation or dephosphorylation to play its role in cell regulation. *YAP* is the encoded product of the *YAP1* gene, which is why *YAP* and *YAP1* are interchangeable terms. Our previous research suggests that intermittent cyclic mechanical tension(ICMT) destroys cellular homeostasis, which causes changes in cartilage calcification-associated genes at the mRNA and protein levels and induces degeneration of the endplate chondrocytes [22–25]. We aimed to determine the relationship of *YAP1* with endplate cartilage degeneration and senescence. Understanding this relationship may be of great significance for the prevention and treatment of endplate cartilage or IDD-related diseases.

## Materials and methods

### Ethics statement and tissue sample acquisition

Human CEP tissue collection and experiments were approved by the Ethics Committee of the First Affiliated Hospital of University of Science and Technology of China (No.: 202109221057000297625) and followed the guidelines of the Helsinki Declaration [26]. All patients were preoperatively examined for disc moisture using magnetic resonance imaging (MRI). The tissue of patients with lumbar disc herniation (LDH;Pfirrmann grades IV–V, n = 15) was surgically obtained using transforaminal NP removal interbody fusion and rod internal fixation. The vertebral tissue in young patients with thoracolumbar vertebral fracture (LVF;Pfirrmann grade I, n = 15) was surgically obtained using open reduction and internal fixation of LVFs (Table 1). To obtain the human CEP tissue, NP and the surrounding soft tissues were carefully dissected under a microscope. The CEP of the LVH group was cut into 1–2mm^3^, washed with phosphate-buffered saline (PBS), and digested with 0.25% type II collagenase (Gibco, USA) at 37 °C for 4 h. The cell suspension was passed through a filter (70 μm pore size), centrifuged at 2000 rpm for 5 min, and resuspended in an F12 medium (Hyclone, USA) containing 15% fetal bovine serum (FBS; Gibco, USA) and 1% penicillin–streptomycin (Gibco, USA). The endplate chondrocytes were cultured in an incubator sustaining 5% CO_2_ at 37 °C.

**Table 1:** Patient demographic data

Parameter	LVF	LDH
Sex ratio (M:F)	(7:8)	(7:8)
Age, mean (range), years	49.33 ± 8.68 (30–59)	59.53 ± 19.82 (34–78)
Pfirrmann grades (number)	I (15)	IV (4) V (11)

### Mechanical loading for endplate chondrocytes

The endplate chondrocytes were seeded into BioFlex six-well plates (Flexcell International Corporation, USA) for multidirectional mechanical loading. The Flexcell FX-5000TM strain loading system used a 0.5 Hz sinusoidal strain with 8%, 12%, and 16% elongation for 3 d, at 8 h/d. The cells were set as the loading group after intermittent tension stimulation by adjusting the softness and stiffness of the bottom wall of the six-pore plate to which endplate chondrocytes adhere. The cells in the control group were cultured normally.

### Detection of cell proliferation and apoptosis

The endplate chondrocytes were seeded into 96-well plates at a density of 5 × 10^3^ cells per well. Then, 10 μL of freshly pretreated CCK-8 (Dojindo Molecular Technologies, Tokyo, Japan) was added to a culture dish containing cells and incubated at 37°°C for 2 h. After seeding, the plates were vortexed for 2 min, and the absorbance of each hole was measured at 450 nm using a spectroscopically scanned multimode Microporous plate reader (Bio-Rad).

Apoptosis was analyzed, and 195 μL annexin V-FITC binding solution (Beyotime Institute of Biotechnology, China) was added into the treated endplate chondrocytes. The cells were gently suspended and mixed with 5 μL annexin V-FITC (Beyotime Institute of Biotechnology, China) with 10 μL PI staining solution and seeded at 25 °C in dark. Finally, the cells were placed in an ice bath and dyed through flow cytometry (BD Biosciences). The apoptosis of endplate chondrocytes was analyzed by FlowJo software (TreeStar).

### Endplate chondrocytes culture and senescence identification

Endplate chondrocytes were cultured in Dulbecco's Modified Eagle's Medium (DMEM)/F12 containing 10% FBS and 1% penicillin/streptomycin. Cells were divided into 8%, 12%, and 16%, and each well was plated with six-well plates. After 12 h of plating, cells were platedagain into six-well plates with a density of 1 × 10^6^ cells per well after 8 h of serum-free DMEM/F12 replacement medium. Cell cycle analysis was performed. Senescence-associated β-galactosidase (SA-β-Gal) activity was measured according to the manufacturer’s instructions. For the cartilage tissue of the endplate, the frozen sections were placed in an oven at 37°°C for 15 min anddried, and tissues were surrounded with a tissue pen and fixed for 30 min with a fixative. For endplate chondrocytes, the cell culture medium was removed and washed with PBS or Hanks' balanced salt solution (HBSS) once; then, 250 μL of 4% paraformaldehyde (PFA) was added andthe mixture was fixed at room temperature for 5 min. The cell fixation solution was removed, and cells were washed with PBS or HBSS three times for 5 min each. PBS or HBSS was removed, and 1 mL of SA-β-Gal staining solution was added to each well. The cells were incubated at 37°°C overnight and sealed with a plastic wrap to prevent the staining solution from evaporating; the endplate chondrocytes expressing SA-β-Gal appeared blue under the microscope.

### Tissue staining and immunohistochemical analysis

The CEP was carefully separated, and the intervertebral disc and annulus fibrous tissue were removed and reformulatedin 4% PFA fixation solution for fixation, dehydration, and decalcification. Then, the CEP was embedded in paraffin and sectioned. Sections were stained with hematoxylin–eosin (HE; SolarBio, China). For immunohistochemical staining, we used quench endogenous peroxidase activity for antigen repair. Subsequently, 40 mL of pH 8 ethylenediaminetetraacetic acid (EDTA) repair solution was poured into the induction oven for heating. Then, the sections were placed with the staining rack into the repair solution. After boiling, the repair was stopped after 2 min. Primary antibodies were inoculated with 3%bovine serum albumin and incubated overnight (*YAP1*, 1:500; Cell Signaling Technology, USA). Sections were incubated with secondary antibodies at room temperature for 2 h. Diaminobenzidine color reagent was added to detect immunoreactivity.

### Western blot analysis

CEP tissue protein and endplate chondrocytes protein samples were separated by sodium dodecyl sulfate–polyacrylamide gel electrophoresis and transferred to apolyvinylidene fluoride (PVDF) membrane. The samples were sealed with 5% skim milk, and the PVDF membrane was placed in the following primary antibodies: *YAP1*(1:2000; Cell Signaling Technology), *Sox9*(1:1000; Affinity), *COL-2A*(1:1000; Abcam), *p53*(1:1000; Abclonal), *p21*(1:1000; Affinity), connective tissue growth factor (CTGF, 1:1000; Affinity), and β-actin (1:1000; Zs-BIO). The PVDF membrane was then placed into the secondary antibody solution and incubated for 1 h. The protein was imaged through a gel imaging system in the presence of an ultra-sensitive luminescent chromogenic solution. Finally, the intensity of the bands was quantified using image grayscale analysis.

### Quantitative RT-PCR

Total RNA was extracted using aTrizol reagent, according to the manufacturer's instructions. A quantitative reverse transcription polymerase chain reaction (qRT-PCR) starter kit (C11030, RiboBio, China) was used to synthesize first-strand cDNA from 2 μg RNA, and the qPCR conditions for the reaction protocol were as follows: stage 1, 95 °C for 5 min; stage 2, 95 °C for 5 s; stage 3, 60 °C for 30 s; and stage 4, 72 °C for 30 s. The relative expression levels of genes were normalized to the value of Gapdh using the ΔΔcycle threshold (ΔΔCq) method. The primer sequences are shown in Table 2.

**Table 2:** Specific primers

Gene	Forward (5′–3′)	Reverse (3′–5′)
β-actin	CCCATCTATGAGGGTTACGC	TTTAATGTCACGCACGATTTC
Collagen II	CAAGAAGGCCTTGCTCATCC	CCATCCTTCAGGGCAGTGTA
Sox-9	TGGAGACTGCTGAACGAGAG	GCCCATTCTTCACCGACTTC
P53	GGGAATGGGTTGGTAGTTGC	TTTCACTGTAGGTGCCAGGT
P21	TTGTGGTAGTTGGAGCTGGT	TGACCTGCTGTGTCGAGAAT
CTGF	CGAAGTGAGAACCGTGTGTC	CTGGCATCTCCACTTCCA
U6	CTCGCTTCGGCAGCACA	AACGCTTCACGAATTTGCGT

### Gene transfection

PGMLV-CMV-Rat_Yap1-EF1-ZsGreen1-T2A-Purowas purchased from Genomeditech (Shanghai, China). The control group and ICMT 16% groups cells were seeded in a six-well plate for 24 h at medium density (1 × 106) before transfection. After 24 h, cells were transfected with PGMLV-CMV-Rat_Yap1 for 36 h, according to the manufacturer’s instructions. Flow cytometry was used to detect the transfection efficiency. After cell transduction was completed, cells were collected for Western blot, qRT-PCR, and follow-up experiments.

### Construction of rat IDD model and grouping

GPAAV-CMV-Rat_Yap1-EF1-ZsGreen1-WPRE was purchased from Genomeditech (Shanghai, China). The experimental procedures of our study were approved by the Ethics Committee of the First Affiliated Hospital of University of Science and Technology of China (Hefei, China) (No.: 202109221037000004850). A total of 40 specific pathogen-free 12–14 week old male rats, weighing 260 ± 15 g, were provided by the Laboratory Animal Experimental Center. The IDD model was produced by needle puncture of rat tail endplate cartilage. The rats were randomly divided into four groups with 10 rats each. The rats in the non-intervention group were labeled as the control group, and the rats in the model group were given intraperitoneal anesthesia. After satisfactory anesthesia, local alcohol disinfection was performed, and an 18G needle was inserted into the C5–C6 or C7–C8 intervertebral discs in a vertical direction with a depth of approximately 3–5 mm. The 18G needle was rotated 360° and held for 30 s; the rats who underwent this procedure were included in the IDD group. For the rats in the non-intervention group, 10 μL of GPAAV-CMV-Rat_*Yap1* was injected into the C5–C6 or C7–C8 endplate cartilage by intraoperative X-ray fluoroscopy with a 26G needle; this was labeled as the Ctrl + AAV-YAP1 group. After puncture at 8w, 10 rats showed disc degeneration on MRI; this was recorded as the IDD + AAV-YAP1 group. MRI was performed 8w after puncture to observe IDD in the four groups, and the Pfirrmann grading system was used for evaluation and comparison. All rats were sacrificed by inhalation of 7.5% isoflurane, and the tail intervertebral disc tissue samples were collected for pathological staining and immunohistochemistry.

### MRI examination and Pfirrmann grade evaluation

After abdominal anesthesia was satisfactory, the rat tails were scanned using 3.0 T MRI. Subsequently, 2-D spin-echo double-echo sequence was performed using the following parameters: repetition time = 9000 ms, flip Angle = 90°, echo time = 16 and 80 ms, slice thickness = 0.6, mean value = 2, field of view = 40 × 40 mm^2^, and 30 sagittal plane resolution = 0.1 mm. The signal intensity of the intervertebral disc was calculated using T2-weighted image (echo time = 80 ms) to monitor intervertebral disc hydration. The signal brightness of the intervertebral disc was used as the reference of the signal intensity of the injured disc in each group. The midsagittal section of the tail was identified for disc degeneration using Pfirrmann Grading [27].

### Histological analysis

After MRI examination, the rats were sacrificed, and the vertebral bodies near C5–C6 and C7–C8 were punctured and fixed with 4% PFA at 4 °C for 24 h, decalcified with 10% EDTA for 30 d, embedded in paraffin, and cut into 5-µm-thick sections. Then, the sections were dehydrated under an ethanol gradient (70–90%) and stained with HE for 30 min at room temperature (Nanjing Jiancheng Bioengineering Institute, Nanjing, Jiangsu, China) according to the manufacturer's instructions.

### Statistical analysis

SPSS 23.0 (Stanford University, Stanford, California) was used for statistical analysis of the data. Data are presented as mean ± standard deviation (SD). The data were evaluated for significant differences using one-way analysis of variance (ANOVA) and compared between the two groups using the Tukey test. *P* < 0.05 was considered statistically significant.

## Results

### The degeneration and senescence of endplate chondrocytes were correlated with the expression of YAP1

**Fig. 1:**
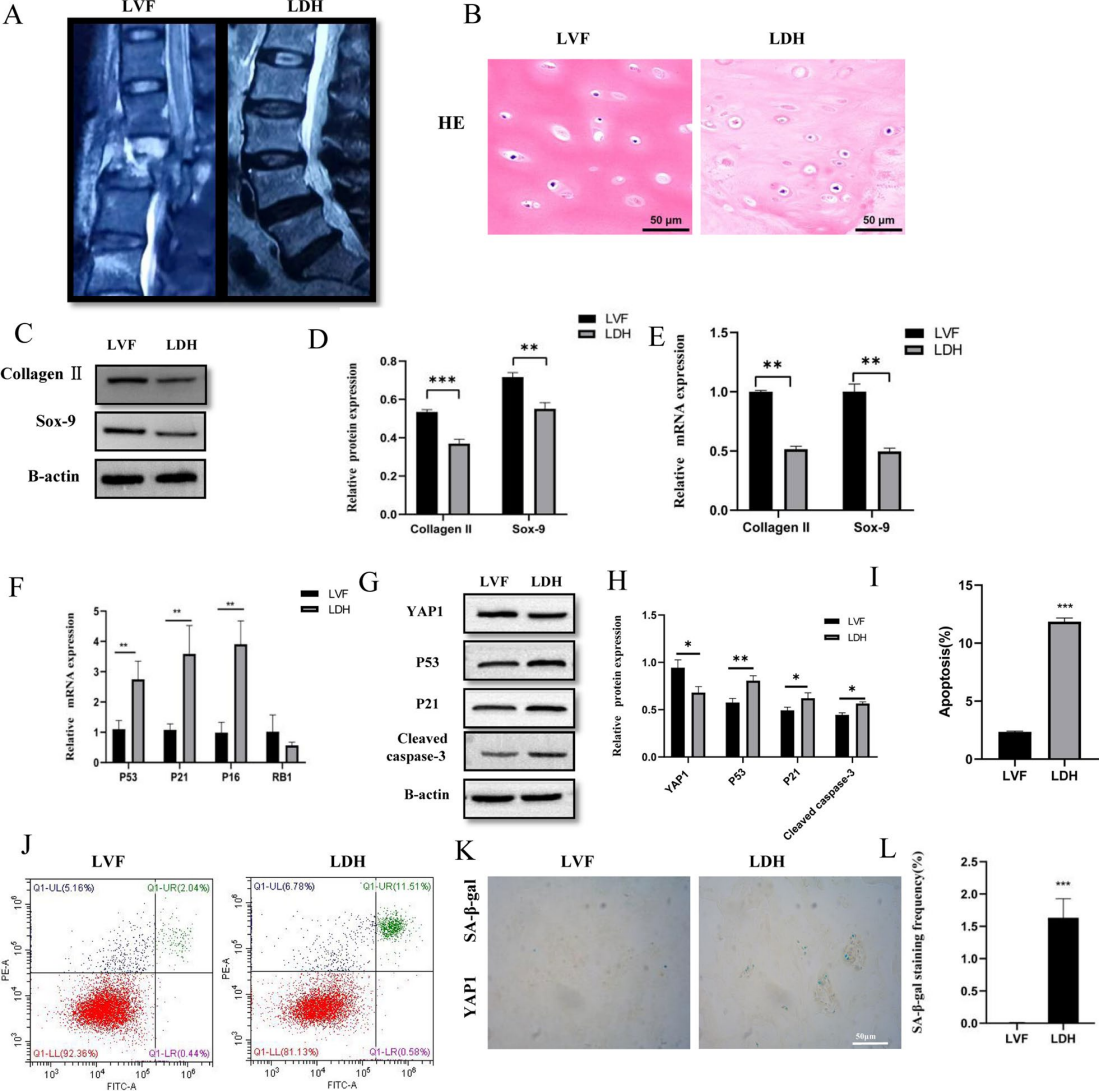
Tissue sample acquisition and analysis. **A** Sagittal magnetic resonance imaging scans LVF and LDH groups. **B** HE staining confirmed that LVF and LDH endplate cartilage had different pathological changes. **C**–**E** Western blotting and RT-qPCR were used to detect the difference of *COL-2A* and *Sox-9* protein expression between the two groups. **F–H** RT-qPCR and western blotting were used to detect the difference in *p53, p21, p16,* and *RB1* expression in the LVF and LDH groups. **I, J** Flow cytometry and **K, L** SA-β-gal staining used to detect the apoptosis and senescence of endplate cartilages respectively (*p* < 0.001)

According to the Pfirrmann grade evaluation classification, 30 human CEP tissues were divided into the LVF and LDH groups (Fig. 1a). HE staining showed that endplate chondrocytes in the endplate cartilage tissue of the LVF group were evenly distributed in the cartilage lacunae, and the cartilage matrix was uniformly stained red (Fig. 1b). The red staining of the cartilage matrix in the LDH group was significantly reduced than that of the LVF group. Then, qRT-PCR and western blot analysis showed that, with the deterioration of CEP, the expression of *COL-2A*, *Sox9*, and *YAP1* decreased (*P* < 0.05) (Fig. 1c–e); conversely, the expression of p53 and p21 increased (*P* < 0.05) (Fig. 1f–h). Additionally, the mean positive percentage of endplate chondrocytes apoptosis (Fig. 1i, j) and SA-β-Gal staining (Fig. 1k, l) were significantly elevated in the LDH group than that of the LVF group (P < 0.05). These results indicated that, with the degeneration of lumbar CEP, the expression of *YAP1* was decreased, and the senescence of endplate chondrocytes was stimulated. This implied that endplate chondrocytes degeneration was associated with cell senescence.

### ICMT promotes endplate chondrocytes degeneration and senescence

**Fig. 2:**
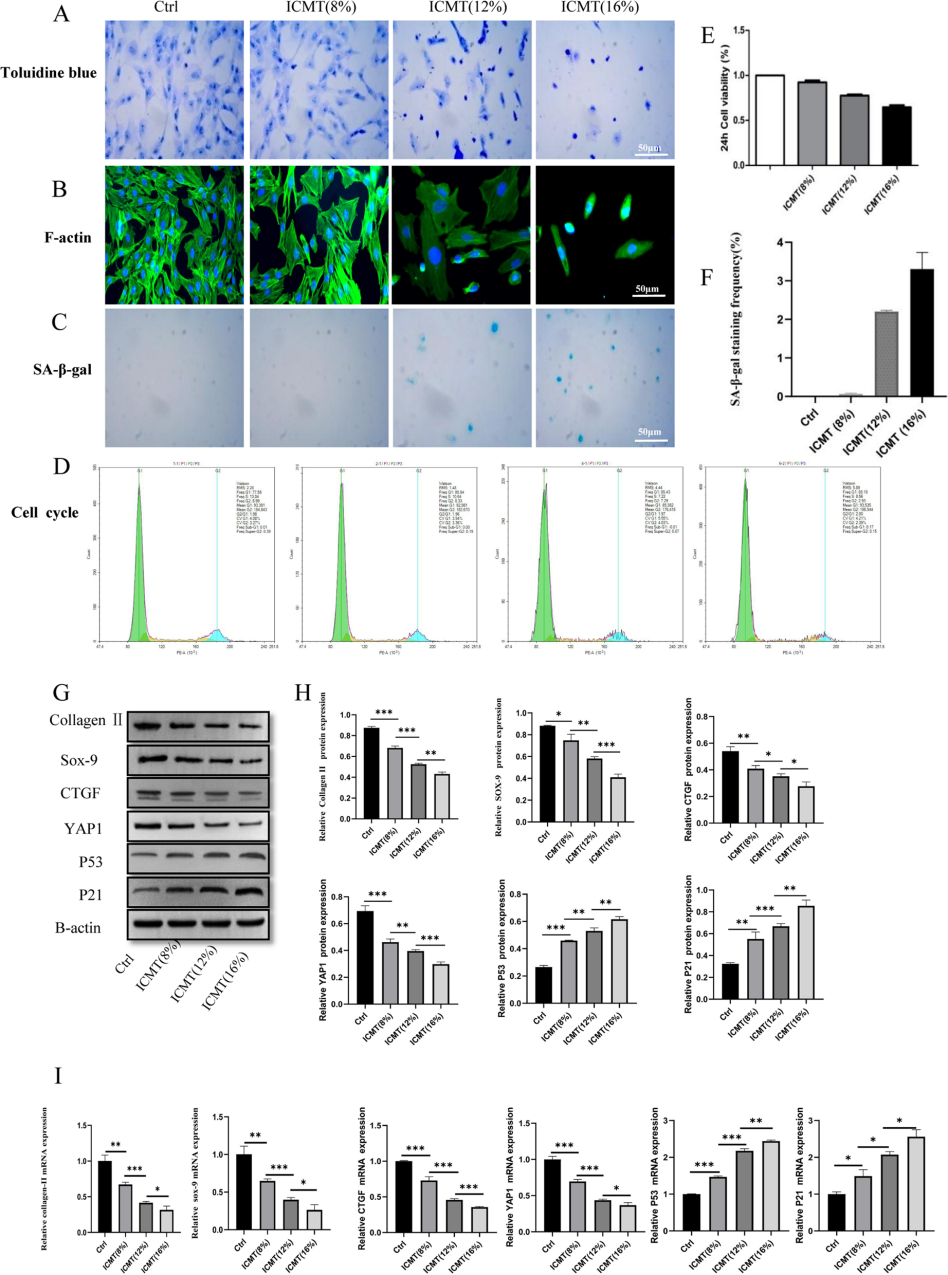
ICMT promotes endplate cartilages degeneration and senescence. **A**, **B** Toluidine bluestaining and F-actin confirmed that Ctrl, ICMT 8%,12%, and 16% endplate chondrocytes had different pathological changes in morphology. **C, D, F** SA-β-gal staining and cell cycle used to detect senescence of endplate chondrocytesin each group. **E** The effect of different tension of ICMT on the proliferation of endplate chondrocytes. **G, H** Western blotting was used to detect the difference in *COL-2A, Sox-9, YAP1, CTGF, p53,* and *p21* protein expression between each group. **I** RT-qPCR was used to detect the difference of *COL-2A, Sox-9, YAP1, CTGF, p53*, and *p21*protein expression between each group

IDD is usually associated with the degeneration of endplate chondrocytes; therefore, we detected the expression of endplate cartilage related genes in patients with lumbar spine fracture (control group). In the ICMT group, the increased duration of tension loading caused significant inhibition in endplate chondrocytes metabolism. Toluidine blue staining showed that the morphology of endplate chondrocytes gradually changed after ICMT stimulation. The increased loading force caused gradual changes in cell shape from oval to long spindle shape; additionally, the cell arrangement in the ICMT 16% group became sparse (Fig. 2a). To determine whether ICMT-induced endplate cartilage degeneration was caused by cell death, we examined the cell viability and F-actin of endplate chondrocytes after exposure to ICMT. These data suggested that ICMT did not promote endplate chondrocytes death but promoted a decrease in endplate chondrocytes viability (Fig. 2e) and re-orientation of the cytoskeleton (Fig. 2b). Furthermore, qRT-PCR and western blot analysis showed reductions in the expression of *COL-2A*, *Sox9*, *YAP1*, and *CTGF* in the endplate cartilage after ICMT loading (Fig. 2g–i). Additionally, these analyses showed increased expression of p53 and p21 after ICMT loading.

### ICMT promotes SA-β-Gal activity and G1 phase

SA-β-gal staining is another important characteristic of senescent cells. As it increases gradually with ICMT, the mean positive percentage of SA-β-Gal staining was significantly elevated in the ICMT 16% group than that of the control group (Fig. 2c, f). As G1 cell cycle arrest is a marker of senescent cells, we analyzed the cell cycle of endplate chondrocytes. Compared to the ICMT 8% group cells, ICMT 16% group cells were easier to arrest in the G1 phase (88.18:80.84) (Fig. 2d). The proportion of G2 and S phases represents the proportion of cell proliferation. ICMT 16% group cells were less frequently observed in G2 and S phases than the control group cells. These results indicated that ICMT not only caused degeneration of endplate chondrocytes but also reduced cell proliferation and stimulated cell senescence.

### Over-expression of YAP1 alleviated ICMT-induced endplate chondrocytes degeneration and senescence

**Fig. 3:**
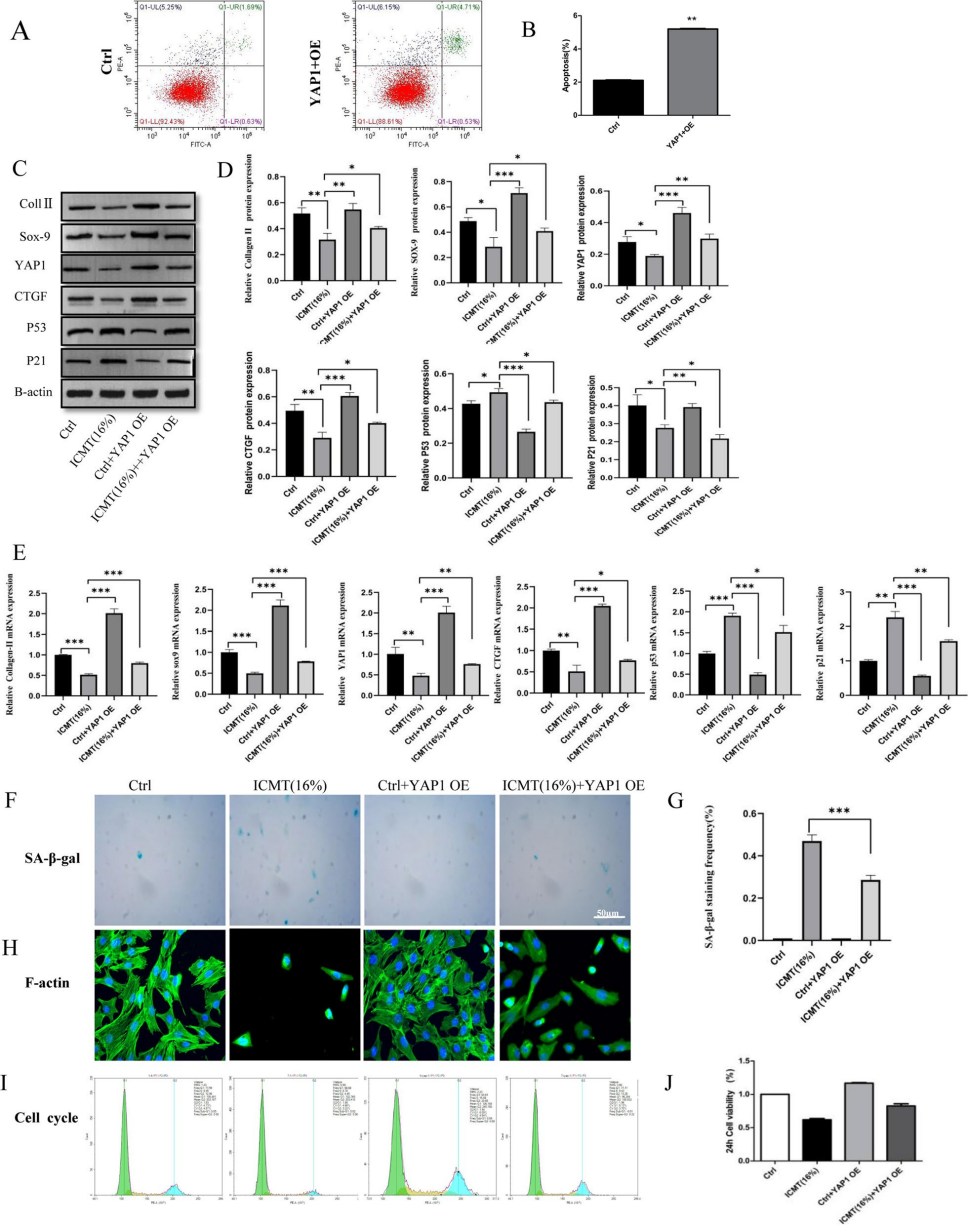
Over-expression of *YAP1* alleviated ICMT-induced endplate chondrocytes degeneration and senescence. **A**, **B** Flow cytometry was used to detect the efficiency of *YAP1* transfection. **C, D** Western blotting was used to detect the difference in *COL-2A, Sox-9, YAP1, CTGF, p53*, and *p21* protein expression between each group. **E** RT-qPCR was used to detect the difference of *COL-2A, Sox-9, YAP1, CTGF, p53*, and *p21*protein expression between each group. **F–H** SA-β-gal staining and F-actin were used to detect the senescence of endplate endplate chondrocytes in each group. **I, J** Cell cycle and cell Counting Kit-8 were used to observe the effect on the proliferation of endplate endplate chondrocytes in the different groups

Flow cytometry was used to detect the transfection efficiency (Fig. 3a, b), which showed an increasing the expression of *YAP1*. Meanwhile, qRT-PCR and western blot analysis showed an increase in the expression of *COL-2A*, *Sox9*, *YAP1*, and *CTGF* in the ICMT 16% group endplate cartilage after cells were transfected with PGMLV-CMV-Rat_Yap1for 36 h; additionally, these analyses showed decreased expression of *p53* and *p21 *(Fig. 3c–e). These results were further confirmed by SA-β-gal staining (Fig. 3f, g), F-actin (Fig. 3h), cell cycle (Fig. 3i), and CCK-8 assay (Fig. 3j). These results suggest that the degeneration and senescence of endplate cartilage are induced by interference with the Hippo pathway through *YAP1* and *CTGF.*

### Over-expression of YAP1 can alleviate IDD

**Fig. 4:**
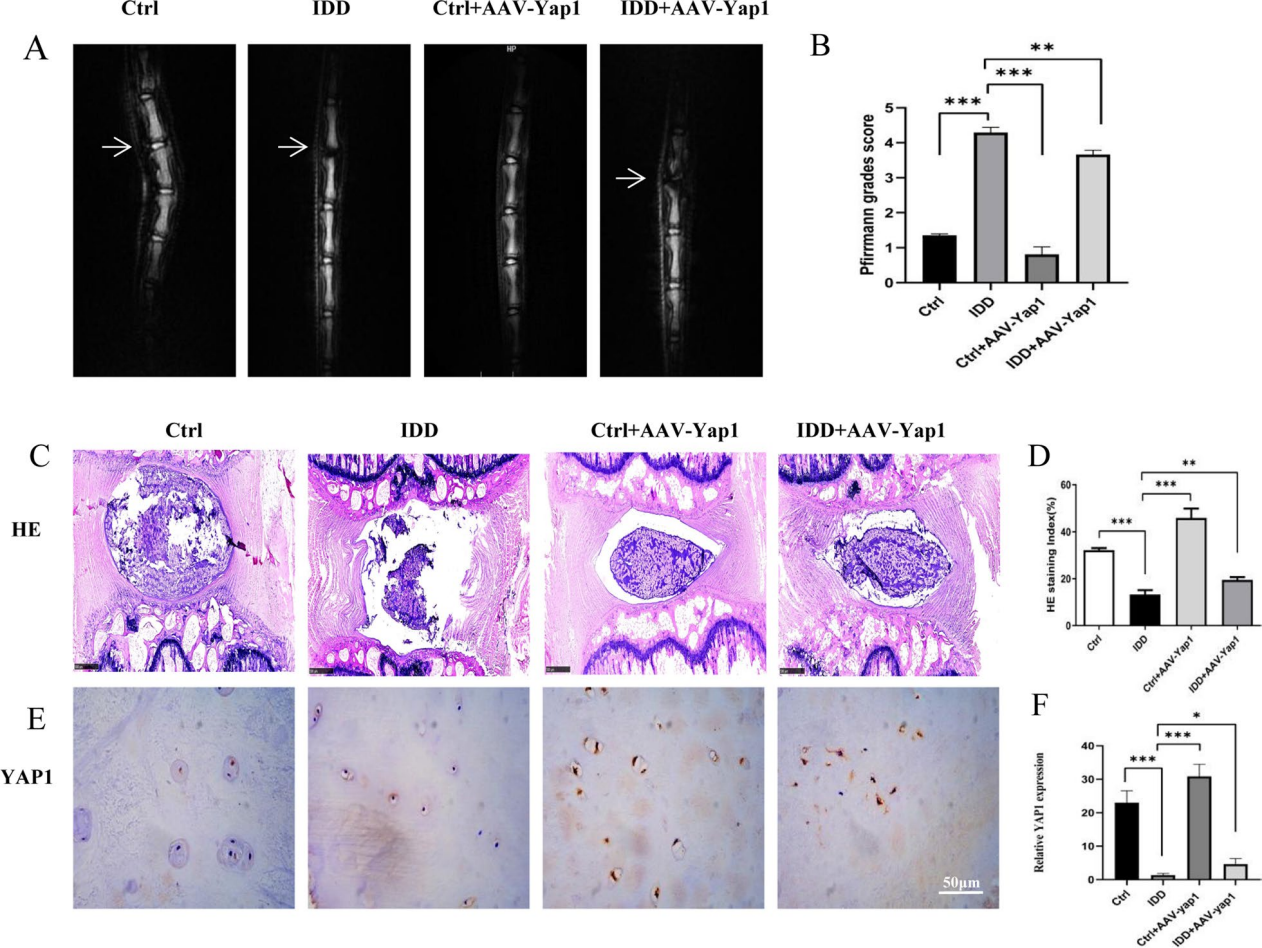
*YAP1* treatment can alleviate IDD in rats. **A, B** IDD in rats determined using magnetic resonance imaging. **C, D** Histological analysis of endplate cartilage and intervertebral disc tissues determined by using H&E staining. **E, F** Immunohistochemistry was used to observe the expression of *YAP1* in rat tail endplate cartilage

Imaging findings of intervertebral discs in rats in the four groups were evaluated using Pfirrmann grading on MRI. After 8 weeks of interference, MRI revealed that the intervertebral disc signal in rats in the control group was normal; in rats in the IDD group, the intervertebral disc signal was significantly reduced. The intervertebral disc signal of rats in IDD + AAV-YAP1 group slightly recovered than that of the IDD group (Fig. 4a, b). HE staining analysis showed that NP and annulus fibrosus in the control group was uniform and with a complete structure (Fig. 4c). The IDD model group showed severe annulus fibrosus disorder and rupture, and the NP tissue disappeared. AAV-YAP1 treatment significantly alleviated the annulus arrangement disorder and protected the cartilaginous and fibrous structures of the disc endplate (Fig. 4c, d). Immunohistochemical analysis indicated that the expression level of *YAP1* was decreased in the IDD model, which was attenuated by AAV-YAP1 injection (Fig. 4e, f). These results suggest that *YAP1* could alleviate degeneration of endplate cartilage in rats in vivo.

## Discussion

The intervertebral disc is the largest avascular tissue in vertebrates; thus, it mainly relies on the adjacent endplate micropores to provide nutrition through diffusion [28]. These endplate micropores are located in the spinal vertebral and NP with a layer of transparent cartilage between. The NP, annulus fibrosus, and CEP are interdependent to maintain their respective integrity. The key factor to maintain stability is the rich proteins in the CEP. Polysaccharides can regulate the transport of materials between the disc and endplate and maintain the protein content of disc. Normal tension load is conducive to this nutrient penetration; however, excessive tension load causes calcification of CEP leading to severe loss of its diffusion ability. Subsequently, this affects the nutrient supply of intervertebral disc cells and lead to IDD [29]. Therefore, degeneration of the CEP is the initiation and key factor of IDD [30]. HE staining demonstrated even distribution of the endplate chondrocytes in the cartilage lacuna, and the cartilage matrix was uniformly stained red in the cartilage tissue of the LVF group endplate. We found degenerated and decreased extracellular matrix in the endplate chondrocytes of patients with LDH than that of patients with LVF. Furthermore, qRT-PCR and western blot showed that the expressions of *COL-2A* and *Sox9* were decreased. These results suggest that CEP degeneration affected IDD progression.

Upstream membrane protein receptors of the Hippo signaling pathway activate *MST1/2* upon receiving extracellular signals; *MST1/2* phosphorylates and interacts with salvador to phosphorylate *LATS1/2* kinase, and the phosphorylated *LATS1/2* kinase is activated. Activated *LATS1/2* kinase inactivates a portion of *YAP1* by phosphorylating downstream Ser127, which is then translocated and sequestered from the nucleus into the cytoplasm and subsequently degraded. Dephosphorylated *YAP1* is activated upon nuclear entry, thereby contributing to the regulation of cell proliferation by driving growth-promoting genes, such as *CTGF* and cysteine-rich angiogenesis inducer 61 (*Cyr61*) through the TEA domain transcription factor family. However, a study of NP tissue examination found that [31] the expression of decreased with age in young rats; conversely, the expression of upstream *LATS1* increased with age. *LATS1* was regulated at the transcriptional level and became a direct target gene of *YAP1*, and *LATS1* level increased with *YAP1* activation [32]. Thus, the regulation of *YAP1* and *LATS1* constitutes a negative feedback loop to ensure that *YAP* overactivation does not occur and Hippo signaling homeostasisis maintained. In our paper, qRT-PCR, western blot, and immunohistochemistry showed that both *YAP1* and *CTGF* in the *Hippo-YAP* signaling pathway were statistically significant, and the expression of the LDH group decreased than that of the LVF group. As an important transcription factor in the Hippo pathway, *YAP1* has been confirmed to be involved in the regulation of various cellular processes, including mechanical stress conduction and stem cell proliferation and differentiation. Regardless of the expression changes of upstream elements, *YAP1* can play a role in regulating cell metabolism, proliferation, and differentiation only through its phosphorylation or dephosphorylation. Therefore, we selected *YAP1* as our research target.

Abnormal mechanical tension is a risk factor for IDD [32]. We observed IDD changes by ICMT, including collagen structure disorder, decreased matrix anabolic metabolism, and decreased cell proliferation ability. An abnormal load increased the number of SA-β-Gal positive cells and up-regulated the expressions of senescence related genes *p16*, *p27*, *RB*, *PTEN*, *p27KIP*, *p19ARF*, and *RAGE* [33], suggesting that abnormal mechanical tension promoted the senescence of intervertebral disc cells [34], which is consistent with the results of our paper. These results provide further evidence that intervertebral disc cell senescence is involved in the pathogenesis of IDD. Regarding the biomarker of cellular senescence, the first one was found to be SA-β-Gal. At pH 6.0, SA-β-Gal activity was associated with lysosomal activity, which was associated with aging. By comparing the two groups of patients, we found that LDH group SA-b-Gal staining was significantly higher than that in the LVF group, and corresponding changes also occurred in the subsequent ICMT and *YAP1* overexpression groups. However, SA-β-Gal is not a specific biomarker of aging because its activity is affected by lysosomal status. Therefore, other molecular biomarkers were used to detect cellular senescence; these biomarkers include *p53*(tumor suppressor gene), cell cycle kinase-dependent (CDK) inhibitors (*p16* and *p21*), cell cycle modulators (retinoblastoma protein, Rb), *p38* and telomere length. Through our verification, the expressions of *p53*, *p21*, and Rb in senescent cells of degenerative lumbar endplate cartilage were increased. This was consistent with literature reports that the p53-p21-Rb pathway was activated in human NP cells, which can induce replicative senescence of intervertebral disc cells [35]. The cell cycle showed similar trends and results. These results indicate that ICMT can not only cause degeneration of endplate chondrocytes, but also reduce cell proliferation and stimulate cell senescence. Furthermore, overexpression of *YAP1* slowed the degeneration and senescence of endplate chondrocytes, which may be another therapeutic approach for IDD.

To further verify the effect of *YAP1* on endplate cartilage degeneration in vivo, an 18G needle was used for tail needle puncture, and a 26G needle was used for injection. According to the MRI examination results of the model group 8 weeks after puncture, the IDD group demonstrated edema and destruction of endplate cartilage and significant reduction of water content and collapse of intervertebral disc. HE staining clearly showed that the NP tissue of the IDD group became sparse, and the annulus fibrosus was disordered. Therefore, the puncture model of rat tail disc degeneration was successful in our group. A local injection dose of 10 μLwas selected for the 12–14-week old rats. After 4 weeks of injection, MRI showed changes in the model group under the interference of AAV + YAP1. T2-weighted imaging showed that, although the IDD remained evident, the intervertebral disc signal was low, becoming slightly gray/black; the intervertebral disc and NP boundary were not clear with intervertebral disc collapse. However, HE also showed that NP tissues in the dyed water slightly increased, and the annulus fibrosus rearranged and recovered in the model group. Immunohistochemistry showed that the expression of *YAP1* was alleviated than that of the IDD group, showing that *YAP1* can delay the senescence and degeneration of rat intervertebral discs by injecting *YAP1* adenovirus into the rat tail intervertebral disc, which may be a new treatment for IDD.

## Conclusion

The degeneration and senescence of endplate chondrocytes were correlated with the expression of *YAP1*. ICMT promoted the senescence and degeneration process of endplate chondrocytes, thereby causing IDD. Thus, over-expressing *YAP1 *in vitro could rescue the senescence and degeneration of endplate chondrocytes induced by ICMT and alleviate IDD.

## Data Availability

According to the requirements, data and materials can be obtained from the corresponding authors to support the results of this study.

## Abbreviations

ANOVA: Analysis of variance
CDK: Cycle kinase-dependent
CTGF: Connective tissue growth factor
FBS: Fetal bovine serum
HBSS: Hanks' balanced salt solution
ICMT: Intermittent cyclic mechanical tension
IDD: Intervertebral disc degeneration
LDH: Lumbar disc herniation
LVF: Lumbar vertebra fracture
MRI: Magnetic resonance imaging
NP: Nucleus pulposus
PBS: Phosphate-buffered saline
SD: Standard deviation
YAP: Yes-associated protein
LBP: Low back pain
SOX-9: SRY-related high-mobility-group-box gene 9
ECM: Extracellular matrix
TGF: Transforming growth factor
CTGF: Connective tissue growth factor
Cyr61: Cysteine-rich 61
MRI: Magnetic resonance imaging
Lats1/2: Large tumor suppressor homologue
Mst1/2: Mammalian Ste20 like protein
